# A mutant Pfu DNA polymerase designed for advanced uracil-excision DNA engineering

**DOI:** 10.1186/1472-6750-10-21

**Published:** 2010-03-16

**Authors:** Morten HH Nørholm

**Affiliations:** 1 Center for Biomembrane Research, Department of Biochemistry and Biophysics, Stockholm University, SE-106 91 Stockholm, Sweden

## Abstract

**Background:**

The combined use of restriction enzymes with PCR has revolutionized molecular cloning, but is inherently restricted by the content of the manipulated DNA sequences. Uracil-excision based cloning is ligase and sequence independent and allows seamless fusion of multiple DNA sequences in simple one-tube reactions, with higher accuracy than overlapping PCR.

**Results:**

Here, the addition of a highly efficient DNA polymerase and a low-background-, large-insertion- compatible site-directed mutagenesis protocol is described, largely expanding the versatility of uracil-excision DNA engineering.

**Conclusions:**

The different uracil-excision based molecular tools that have been developed in an open-source fashion, constitute a comprehensive, yet simple and inexpensive toolkit for any need in molecular cloning.

## Background

**Figure 1:**
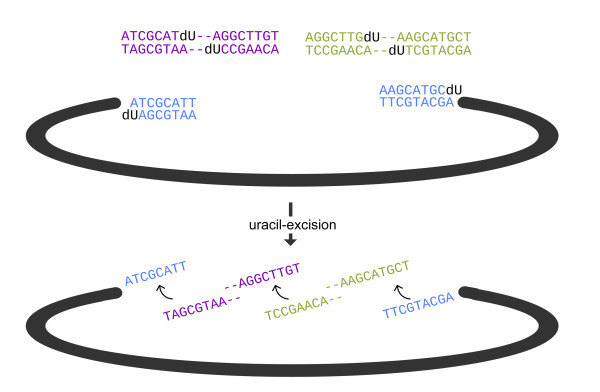
**Principle of uracil-excision based DNA fusion**. In uracil-excision based DNA fusions, dU nucleotides replace selected thymidine nucleotides in DNA and is subsequently removed by e.g. the USER™ enzyme, leaving the upstream nucleotide sequence unstable. When the resulting, compatible single stranded overhangs are combined they can be transformed directly into bacteria without prior ligation to yield a stable recombinant molecule.

Uracil-excision-based cloning was invented more than 15 years ago [1,2], but the technique was left unused, due to incompatibility with high-fidelity PCR [3,4]. In the technique, compatible single stranded DNA overhangs are created by substituting selected deoxy thymidine (dT) nucleotides with deoxy uridine nucleotides (dU) (Figure 1). Subsequently, the DNA is treated with uracil DNA glycosidase (UNG) and usually either T4 endonuclease [5] or DNA glycosylase-lyase endo VIII (commercially available as a mix with UNG as the so-called USER™ enzyme from New England Biolabs), which releases the sequence upstream from the dU's and allows pairing between exposed, compatible ends (and these reactions define the term "uracil-excision" as it is used here). The overhangs are usually designed to be 7-12 nucleotides long, and therefore can create circular DNA species that are stable enough to allow bacterial transformation without prior ligation. Hence, the technique relies solely on a pair of properly spaced deoxy adenine- and dT-nucleotides, and, due to the degeneracy of the genetic code, allow seamless translational fusions of virtually any protein coding DNA sequence, making it particular suitable for (but not limited to) applications such as chimeric DNA fusion designs.

In 1999, the crystal structure of the DNA polymerase Tgo from the archea *Thermococcus gorgonarius *was solved [6], revealing the nature of the uracil-binding pocket, and allowing the design of mutant Tgo- and Pfu-polymerases with reduced stalling at uracil-containing DNA [7]. With mutants like PfuV93Q, high-fidelity PCR became compatible with uracil-excision cloning. This led to development of an improved uracil-excision cloning technology [3] and to a new way of doing seamless PCR product fusion that may eventually replace overlapping PCR in many applications [8,9]. The useful application of these technologies was further demonstrated by their use in artificial gene synthesis [10].

In addition to PCR, and cloning and fusing genes, site-directed mutagenesis is an indispensable tool for molecular biologists. One of the early methods for doing site-directed mutagenesis was the Kunkel method [11] that uses template DNA isolated from a *ung- dut- E. coli *strain. This strain lacks dUTPase and uracil deglycosidase and therefore accumulates soluble dUTP and DNA-bound dU nucleotides. In the method, dU-containing DNA is used as template in a linear amplification reaction with a mutagenic primer, as well as the Klenow enzyme, dNTPs and a ligase. Subsequently, the DNA is transformed into *ung+ *bacteria, where only the newly synthesized, mutant DNA survives and the primer-introduced mutation is isolated. Later, PCR entered the scene and variants, known as inverse PCR or whole plasmid synthesis (WHOPS), largely seems to have replaced the Kunkel method. In the typical PCR-based approach, template carry-over is inhibited by treatment with the restriction enzyme DpnI, that restricts *dam *methylated plasmid DNA, but leaves unmethylated PCR-derived DNA intact.

**Figure 2:**
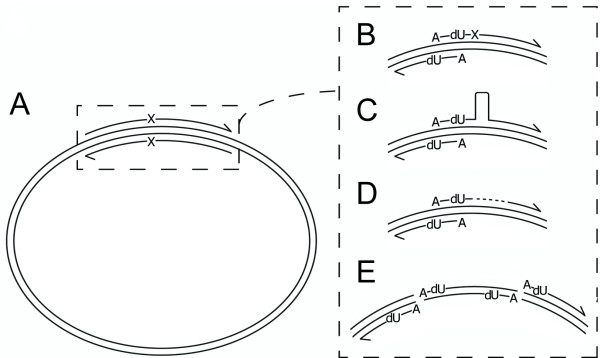
**Principle of whole plasmid synthesis (WHOPS) and uracil-excision based site-directed mutagenesis (U-SM)**. **(A) **In WHOPS, two usually perfectly overlapping oligonucleotides are used to amplify an entire plasmid. WHOPS is used for site-directed mutagenesis by placing mutations (illustrated with an X) in the middle of the two primers. **(B) **Oligonucleotides for U-SM need only to overlap in the complementary region between the selected A- and T-nucleotides and only one primer needs to carry the mutation. **(C) **U-SM is compatible with making insertions (illustrated with a loop) and **(D) **deletions (missing sequence illustrated with a dashed line). **(E) **Larger insertions, such as in e.g. whole gene fusions, are made by a simple combination of the U-SM principle and uracil-excision based cloning - or even multiple fragments (not shown) as in the uracil-excision based PCR fusion principle.

Uracil-excision can be used in several ways as a simple, versatile and large-insertion compatible site-mutagenesis WHOPS method. The method offers several advantages compared to previous WHOPS protocols (Figure 2A-E). First, whereas traditional exponential amplification WHOPS uses two unique primers for each mutation (Figure 2A), uracil-excision offers the opportunity to reuse one primer when several mutations need to be introduced in one location (Figure 2B). Second, the primers do not to overlap in their 3'-end, and, hence, are shorter and should perform better in PCR (Figure 2B). Third, given the limitation in DNA-synthesis chemistry, the reduced size of the primers offers the opportunity to create larger insertion mutations (Figure 2C) and offers the same possibilities with deletions (Figure 2D). Fourth, uracil-excision can be used for very large insertions (Figure 2E, [4,8,9]), free from the inherent limitations in overlapping PCR. However, most importantly, the protocol is highly versatile and insertions can be created from a variety of DNA species, ranging from small, perfectly complementary DNA oligonucleotides, over medium-range DNA species created by enzymatic extension of oligonucleotides that only overlap in their 3'end, to DNA of virtually any length created by PCR - and several different approaches can be combined in one-tube format to introduce various changes in one reaction.

Here, uracil-excision-based artificial gene synthesis is used to create a combination of a PfuV93Q mutant [7] and a highly processive Pfu-SSo7d fusion polymerase [12]. This new DNA-polymerase has several properties that make it uniquely suitable for several of the described applications, including site-directed mutagenesis of large plasmids, and a combination of the modern WHOPS mutagenesis and the classical Kunkel-method to avoid carry-over of template DNA.

## Results

### Fusing the DNA-binding protein Sso7d to PfuV93Q yields a highly efficient polymerase for use in uracil-excision DNA engineering

**Figure 3:**
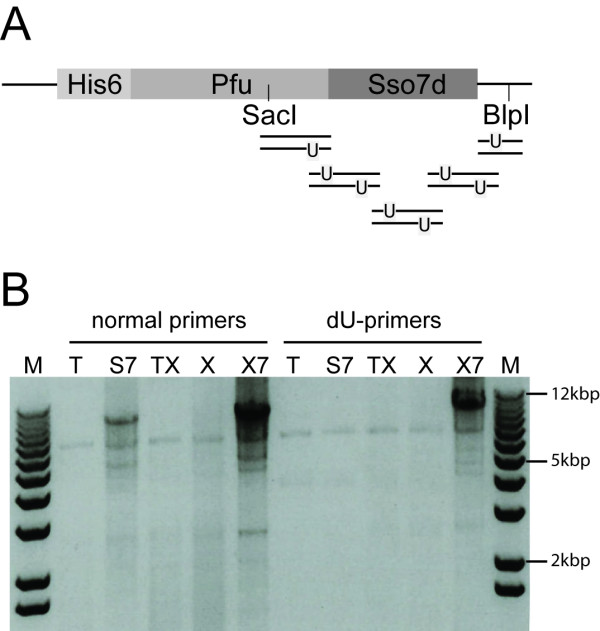
**A new Pfu-sso7d fusion DNA polymerase that is compatible with uracil-excision cloning**. **(A) **Illustration of the modular structure of the Pfu-sso7d DNA polymerase and the oligonucleotides used to fuse the Sso7d gene to Pfu. **(B) **Agarose gel electrophoresis of whole plasmid synthesis PCRs performed with five different Pfu-based DNA polymerases and either standard (normal) oligonucleotides or dU-containing primers. The five different DNA polymerases are PfuTurbo (T), Phusion (S7), PfuTurboCX (TX), Pfu-(V93Q) (X) or PfuX7 (×7) and the molecular marker (M) is kb+ (Invitrogen).

One of the drawbacks of uracil-excision molecular cloning and PCR fusion, is that it is only compatible with two different, commercially available DNA polymerases: the non-proof-reading Taq polymerase (Taq) and the proof-reading polymerase PfuCX (Stratagene), both which suffer from having low processivity [12]. Recently, the processivity of both Taq and Pfu was greatly improved by fusing the enzymes to the small DNA-binding protein Sso7d (yielding S7-Taq and PfuS7), derived from the thermophilic archea *Sulfolobus solfataricus *[12]. In our lab, it has been found that the commercial version of the Pfu fusion protein (Phusion, Finnzymes), performs significantly better in WHOPS of large (5-15 kb) plasmids. Hence, the uracil-binding pocket mutation (V93Q) version of PfuS7 will greatly improve the performance of the uracil-excision toolkit, in particular in challenging PCR reactions such as site-directed mutagenesis on large plasmids. A V93Q version of Pfu was first created in a pET-expression vector system by standard WHOPS. Subsequently, due to the lack of an Sso7d DNA template, a uracil-excision based artificial gene synthesis strategy was employed to add an artificial Sso7d gene to the 3'end of the PfuV93Q expression construct (Figure 3A and Additional file 1). This example demonstrates that uracil-excision technology can be used to create entirely artificial genes and this approach is compatible in one-tube format with the other uracil-excision applications such as PCR fragment cloning and site-directed mutagenesis, expanding the versatility of the technology. Using standard Pfu polymerase conditions, we next compared the performance of the purified PfuV93Q-S7 enzyme (termed PfuX7) with the commercial PfuTurbo (PfuT, Stratagene), PfuS7 (Phusion), the commercial PfuV93Q-Turbo (PfuCX, Stratagene), and PfuV93Q using both standard and uracil-containing primers, in a challenging mutagenesis reaction, on a large (approximately 10 kb) E. coli-yeast shuttle vector pTEF423 [13] harboring the plant gene *CRE1 *[14]. The two nearly identical primer pairs were designed to fit either a uracil-excision strategy or a very similar WHOPS strategy [15], and used to introduce a total of 12 mutations in 5 neighboring codons in the *CRE1 *sequence. Standard 1% agarose gel electrophoresis analysis of the products showed that the two Sso7d fusion polymerases were able to amplify the plasmid with standard primers, whereas only PfuX7 amplified the plasmid with uracil-containing primers (Figure 3B). Various conditions were tried, including cycling conditions with very long extension times, but it was never possible to amplify the plasmid with a non-Sso7d polymerase.

### PfuX7 polymerase is compatible with the Kunkel-method to limit carry-over of unwanted template DNA

**Figure 4:**
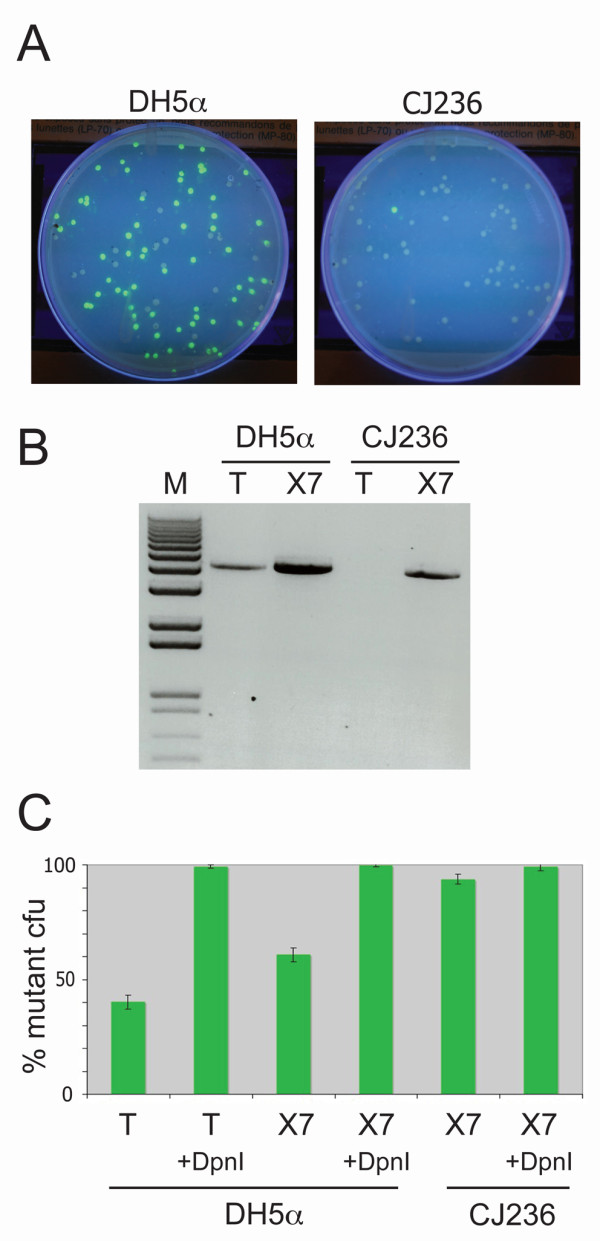
**PfuX7 polymerase is compatible with the Kunkel method for template elimination in site-directed mutagenesis**. **(A) **Screen designed to calculate the efficiency of site-directed mutagenesis. By introducing a stop codon mutation in a plasmid responsible for constitutive expression of the green fluorescent protein in *E. coli *cells, the efficiency of the mutagenesis is easily calculated as the ratio of non-fluorescent cells to total amount of cells. Shown, is a typical example of an experiment using template DNA isolated from either DH5α or CJ236. **(B) **Agarose gel electrophoresis of PCRs performed with the PfuTurbo- (T) or the PfuX7 (×7) DNA polymerases using template plasmid DNA isolated from the *ung+ E. coli *strain DH5α or the *ung- *strain CJ236. The molecular marker (M) is kb+ (Invitrogen). **(C) **Quantification of the efficiency of site-directed mutagenesis using the PfuTurbo- or the PfuX7 DNA polymerases using template plasmid DNA isolated from the *ung+ E*. coli strain DH5α or the *ung- *strain CJ236, with or without DpnI treatment. Data represents the average of three independent experiments with standard deviations.

Previously, two methods have been used to limit template carry-over in site-directed mutagenesis reactions. In the classical Kunkel-method, the template is isolated from a *ung- dut- *strain of E. coli such as CH236 (New England Biolabs), which makes it incompatible with re-transformation into normal *ung+ *bacteria. Exponential WHOPS mutagenesis takes advantage of the DpnI restriction enzyme to restrict methylated template DNA. The combined use of dU-containing DNA with DpnI-restriction was previously demonstrated with a linear amplification mutagenesis strategy [16], but the PfuV93Q versions allow PCR-based site-directed mutagenesis to be combined with both template elimination methods and hence, will greatly decrease the recovery of unwanted template DNA. A simple screen was designed to test this idea, based on a single nucleotide mutation of the green fluorescent protein turning the codon coding for Tyrosine 40 into a TAG stop codon. In the screen, mutants are easily identified as non-fluorescent colonies (Figure 4A). A GFP expression vector [17] was isolated from both CJ236 and the common *ung+ *cloning strain DH5α and used as template in a common 13-cycle protocol with Pfu-Turbo or PfuX7. As expected, only PfuX7 was able to amplify DNA isolated from the CJ236 strain (Figure 4B). Similar results were obtained comparing PfuX7 to Phusion/PfuS7 polymerase (see Additional file 2). Next, aliquots of the reactions were treated with DpnI and the DpnI-treated and untreated reactions were transformed into the BL21 strain. The screen confirmed that very few template-containing colonies were isolated from reactions based on DNA isolated from CJ236, whereas a large proportion of the colonies formed from the DH5α-DNA based reactions exhibited the wildtype, fluorescent phenotype (Figure 4A). Quantifications of the results showed that in all cases, using DNA from CJ236 or DpnI-treatment, resulted in more than 90% mutant recovery (Figure 4C).

### PfuX7 allows the use of dUTP instead of dTTP in PCR

**Figure 5:**
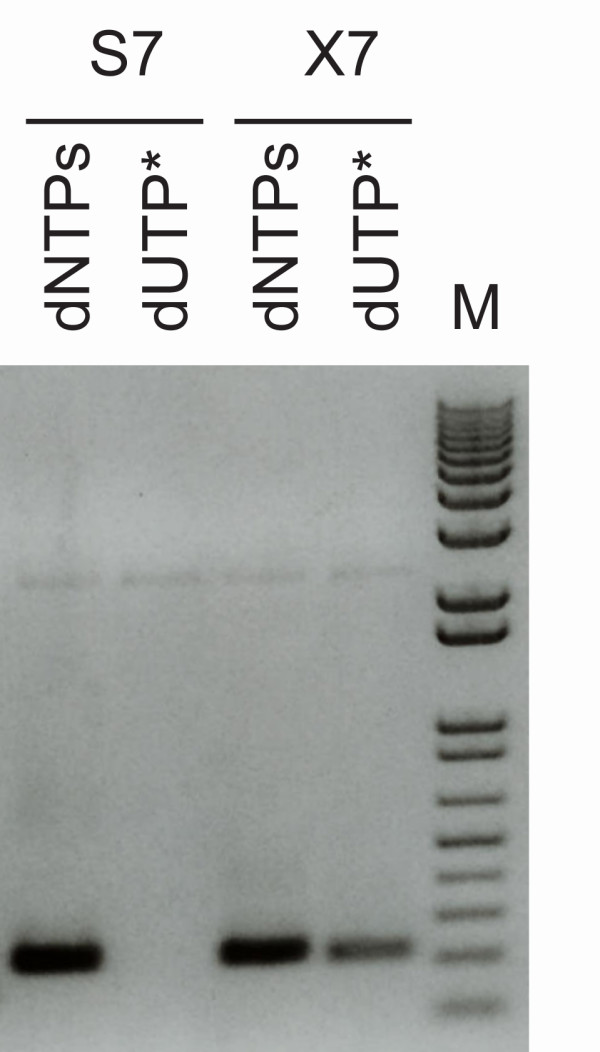
**PfuX7 accepts dUTP in place of dTTP in PCR**. Agarose gel electrophoresis of PCRs performed with the Phusion (S7) or the PfuX7 (×7) DNA polymerases and either standard dNTPs or with a dUTP/dNTP mix where dUTP replaces dTTP. The molecular marker (M) is kb+ (Invitrogen).

PCR revolutionized molecular cloning and largely defined the field of forensic genetics and allowed the analysis of ancient DNA samples of low quality and quantity. However, the efficiency of PCR creates a risk of contamination with previous samples and is of particular concern in e.g. forensic genetics. Several methods have been developed to prevent PCR carry-over contamination. In a widely used method, dUTP replaces dTTP in PCR, and prior to a new reaction UNG-treatment destroys all dU-containing, contaminating DNA species [18]. PfuX7 accepts the replacement of dTTP with dUTP in PCR, and therefore is compatible with UNG-based carry over contamination prevention. This was demonstrated with a standard PCR, with or without dTTP replaced by dUTP. In the experiment, PfuX7 was compared to the other highly processive Phusion polymerase using standard Phusion PCR conditions. Agarose gel electrophoresis confirmed that only PfuX7 was able to perform PCR with dUTP instead of dTTP (Figure 5).

## Discussion

In one of the early PCR-based mutagenesis protocols, WHOPS was performed with non-overlapping phosphorylated primers and was followed by a ligation step prior to transformation of the mutant DNA [15] - the method became available as the Exsite™ PCR-based Site-Directed Mutagenesis Kit (Stratagene) and a similar product is today available as the Phusion™ Site-directed Mutagenesis Kit (Finnzymes). The uracil-excision site-directed mutagenesis approach is very similar. However, compared to the latter technique, ligation is not necessary and the uracil-defined overlaps add an extra quality insurance step since erroneous mismatching extensions on the amplified plasmid will not yield stable recombinant molecules [8]. In the older approach, any phosphorylated DNA species can be ligated to create a circular recombinant molecule.

Today, probably the most widely used technique is WHOPS with a perfectly overlapping primer pair that negates the need for ligation prior to transformation - commercially available from e.g. Stratagene as the QuikChange^® ^Site-Directed Mutagenesis Kit. Due to the exponential nature of WHOPS, and the availability of modern high-fidelity DNA polymerases, such as the highly processive Pfu-Sso7d fusion protein DNA polymerase [12], this is a highly efficient technology, but the technique is not as versatile as uracil-excision.

In site-directed mutagenesis, the DpnI template removal step adds extra time to the procedure and template carry-over is an error frequently observed in our lab. PfuX7 allows the combined use of DpnI-treatment and dU-containing DNA to avoid carry-over of template. In our experimental setup, both the Kunkel-method and the use of DpnI was more than 90% efficient in the prevention of template recovery, and it was therefore not possible to detect a large effect of the combined use. However, in a linear amplification mutagenesis strategy, the combined use of DpnI and dU-DNA was previously reported to increase the efficiency of a mutagenesis from 38% (DpnI alone) to 70-91% [16]. Therefore, the Kunkel-approach to limit tempate carry-over could be a useful and time-saving alternative or addition to DpnI-treatment, in site-directed mutagenesis.

Pfu-Sso7d fusion polymerases, such as Phusion, are marketed as polymerases for direct PCR on complex samples such as blood. Indeed, we have found that in some cases PfuX7 is the only DNA polymerase that produces a PCR product in combination with DNA isolated from e.g. plant material (Nour-Eldin, H. H., personal communication), human cDNA (Lange, J. B., personal communication) or when doing WHOPS on large plasmid DNA templates (this work). Forensic PCR deals with complex samples of low quantity and quality, and prevention of carry-over contamination from previous PCRs is important to this field. PfuX7 polymerase performs better than other DNA polymerases in virtually all applications in our laboratory. Furthermore, none of the wildtype Pfu-based versions (including Phusion) have ever been able to amplify dU-containing DNA. Hence, PfuX7 polymerase may be useful in forensic PCR, both due to its high performance as well as compatibility with the well-established UNG-method for prevention of carry-over contamination.

## Conclusions

The combination of the USER enzyme and the new PfuX7 polymerase described here, constitutes a simple, yet comprehensive molecular toolkit that enables one-tube, ligase-free cloning [3], easy conversion or design of compatible vectors [3], seamless PCR fusions [8,9], artificial gene synthesis [10], site-directed mutagenesis and prevention of carry-over contamination. No other single molecular cloning technology exhibits a comparable versatility.

## Methods

### Poly chain reactions

For comparison of the performance of different Pfu polymerase versions, a 30-cycle PCR was performed with a final concentration of 10 ng/μl pTEF423-*CRE1 *plasmid (a kind gift from Prof. Tatsuo Kakimoto) with a dU-containing mutagenic primer pair Cre1YILY-1UF and Cre1YILY-1UR or a phosphorylated primer pair Cre1YILY-1XF and Cre1YILY-1XR (Table 1), all at a final concentration of 0.5 μM. Cycling conditions included a 30 second 96°C template denaturation step, a 30 second 58°C annealing step and a 5 minute and 30 second long primer extension step at 72°C. The reaction was performed with a final concentration of 0.4 mM dATP, 0.4 mM dTTP, 0.4 mM dCTP, 0.4 mM dGTP, 20 mM Tris/HCl pH 8.8, 10 mM KCl, 6 mM (NH_4_)_2_SO_4_, 2 mM MgSO_4_, 0.1 mg/ml BSA and 0.1% Triton X-100. PCR with dUTP replacing dTTP was performed using standard Phusion (Finnzymes) reaction conditions as recommended by the manufacturer, using the dUTP/dNTPs mix (Fermentas) instead of dNTPs. The pET-Pfu-vector was a kind gift from Jens Lykke-Andersen. The QuikChange^® ^Site-Directed Mutagenesis Kit (Stratagene) was used to introduce the V93Q mutation in pET-Pfu, with the primers Pfu-V93Q-F and Pfu-V93Q-R, and a stop codon in GFP with the primers GFP-Y40STOP-F and GFP-Y40STOP-R (Table 1).

**Table 1:** Oligonucleotides used in this work

*Oligonucleotide*	*Sequence (5'-3')*
Cre1YILY-1UF	ATTGGTTTCdUTGGTGTATATACTGTATGGTGGTGCAGCTATGCACATAGTAAAAGTCGAAGATGATTT
Cre1YILY-1UR	AGAAACCAAdUCGCAAAGAACAATGG
Cre1YILY-1XF	PO_4_TGGTGTATATACTGTATGGTGGTGCAGCTATGCACATAGTAAAAGTCGAAGATGATTT
Cre1YILY-1XR	PO_4_-AGAAACCAATCGCAAAGAACAATGG
Pfu-V93Q-F	GTGGAAACTTTATTTGGAACATCCCCAAGATCAGCCCACTATTAGAGAAAAAGT
Pfu-V93Q-R	ACTTTTTCTCTAATAGTGGGCTGATCTTGGGGATGTTCCAAATAAAGTTTCCAC
Pfu-upstream-F	GCGTTACTGCCTGGGGAAGA
Pfu-R	ACCGCCGGdUACCGGATTTTTTAATGTTAAGCCAGGAAG
Pfu-F	AGCAGAAAAAGdUAGCAATTCGTCGTGCATCTGTTTG
Pfu-downstream-R	GGCCCCAAGGGGTTATGCTA
Sso7d-1F	ACCGGCGGdUGGCGGTGCAACCGTAAAGTTCAAGTACAAAGGCGAAGAAAAAGAGGTAGACATCTCCAAGATCAAGAAAGT
Sso7d-1R	ACTTTCTTGAdUCTTGGAGATGTCTACCTCTTTTTCTTCGCCTTTGTACTTGAACTTTACGGTTGCACCGCCACCGCCGGT
Sso7d-2F	ATCAAGAAAGdUATGGCGTGTGGGCAAGATGATCTCCTTCACCTACGACGAGGGCGGTGGCAAGACCGGCCGT
Sso7d-2R	ACGGCCGGTCdUTGCCACCGCCCTCGTCGTAGGTGAAGGAGATCATCTTGCCCACACGCCATACTTTCTTGAT
Sso7d-3F	AGACCGGCCGdUGGTGCGGTAAGCGAAAAGGACGCGCCGAAGGAGCTGCTGCAGATGCTGGAGAAGCAGAAAAAGT
Sso7d-3R	ACTTTTTCTGCdUTCTCCAGCATCTGCAGCAGCTCCTTCGGCGCGTCCTTTTCGCTTACCGCACCACGGCCGGTCT
GFP-Y40STOP-F	GGGTGAAGGTGATGCTACATAAGGAAAGCTTACC
GFP-Y40STOP-R	GGTAAGCTTTCCTTATGTAGCATCACCTTCACCC

### Uracil-excision-based artificial gene synthesis

Sso7d was fused to the C-terminus of PfuV93Q by amplifying part of the sequence upstream from the fusion site with the primers Pfu-upstream-F and Pfu-R, and part of the sequence downstream from the fusion site with the primers Pfu-F and Pfu-downstream-F (Table 1 and Figure 3A). Next, PCR products were mixed with varied concentrations of three pairs of complementary oligonucleotides (Sso7d-1F + Sso7d-1R, Sso7d-2F + Sso7d-2R and Sso7d-3F + Sso7d-3R, Table 1) and treated with the USER enzyme (New England Biolabs, NEB). Fragments were ligated using the Quick ligase kit (NEB), gel purified the product using the Qiagen gel purification kit and finally used as template in a standard Phusion PCR with the primers Pfu-upstream-F and Pfu-downstream-R. The resulting PCR products and the pET-Pfu vector was treated with the SacI- and BlpI-Fastdigest restriction enzymes (Fermentas) and subsequently fragments of the expected size were gel purified as described above. Finally, the Pfu-Sso7d fragment was ligated into the pET-Pfu-(V93Q) vector with the Quick ligase kit and transformed into a standard *E. coli *cloning strain. The sequence of the Pfu-Sso7d fusion (termed PfuX7) was confirmed by standard DNA sequencing.

### Expression and purification of the PfuX7 polymerase

The pET-PfuV93Q and pET-PfuX7 plasmids were transformed into BL21 cells and transformants were inoculated in LB with ampicillin (50 μg/ml) overnight (ON). An ON culture was back diluted to an optical density of 0.1 at 600 nm and grown to OD 0.3, before protein expression was induced with 0.5 mM isopropyl β-D-1-thiogalactopyranosid for three hours. The culture was then centrifuged at 15.000 g for 10 minutes, the supernatant discarded and the pellet stored at -80°C ON. The his- tagged protein was purified under native conditions using the Qiagen Ni-NTA Spin Kit according to the manufacturers prescriptions. The protein was desalted on Vivaspin 20 columns (Sartorius) columns and stored at -20°C in a solution of 50% glycerol, 100 mM Tris/HCl pH 8.0, 0.2 mM EDTA, 2 mM DTT, 0.2% NP40, 0.2% Tween 20. The protein activity was tested in a dilution series under standard Pfu PCR conditions (as described above) to obtain the optimal performance.

## Supplementary Material

Additional file 1 **Nucleotide sequences of PfuX7 and the oligonucleotides creating the sso7d gene**. Contains the details of the nucleotide sequence of PfuX7 in pdf format. Click here for file

Additional file 2 **Comparison of the DNA polymerases PfuTurbo, Phusion and PfuX7 in a site-directed mutagenesis PCR**. Shows the comparison of PfuTurbo, Phusion (PfuS7) and PfuX7 in a site-mutagenesis PCR in pdf format. Click here for file
