# Supplementary material for: A mutant Pfu DNA polymerase designed for advanced uracil-excision DNA engineering
Source: BMC Biotechnol. 2010 Mar 16;10:21. doi: 10.1186/1472-6750-10-21 (PMC2847956; doi:10.1186/1472-6750-10-21)
Supplement: Additional file 1 — Nucleotide sequences of PfuX7 and the oligonucleotides creating the sso7d gene. Contains the details of the nucleotide sequence of PfuX7 in pdf format. [file 1472-6750-10-21-S1.PDF]

Additional file 1: Details of the nucleotide sequence of PfuX7

Coding sequence:

ATGCATCACCATCACCATCACGGATCAATGATTTTAGATGTGGATTACATAACTGAA
GAAGGAAAACCTGTTATTAGGCTATTCAAAAAAGAGAACGGAAAATTTAAGATAGAG
CATGATAGAACTTTTAGACCATACATTTACGCTCTTCTCAGGGATGATTCAAAGATT
GAAGAAGTTAAGAAAATAACGGGGGAAAGGCATGGAAAGATTGTGAGAATTGTTGAT
GTAGAGAAGGTTGAGAAAAAGTTTCTCGGCAAGCCTATTACCGTGTGGAAACTTTAT
TTGGAACATCCCCAAGATGTTCCCACTATTAGAGAAAAAGTTAGAGAACATCCAGCA
GTTGTGGACATCTTCGAATACGATATTCCATTTGCAAAGAGATACCTCATCGACAAA
GGCCTAATACCAATGGAGGGGGAAGAAGAGCTAAAGATTCTTGCCTTCGATATAGAA
ACCCTCTATCACGAAGGAGAAGAGTTTGGAAAAGGCCCAATTATAATGATTAGTTAT
GCAGATGAAAATGAAGCAAAGGTGATTACTTGGAAAAACATAGATCTTCCATACGTT
GAGGTTGTATCAAGCGAGAGAGAGATGATAAAGAGATTTCTCAGGATTATCAGGGAG
AAGGATCCTGACATTATAGTTACTTATAATGGAGACTCATTCGACTTCCCATATTTA
GCGAAAAGGGCAGAAAAACTTGGGATTAAATTAACCATTGGAAGAGATGGAAGCGAG
CCCAAGATGCAGAGAATAGGCGATATGACGGCTGTAGAAGTCAAGGGAAGAATACAT
TTCGACTTGTATCATGTAATAACAAGGACAATAAATCTCCCAACATACACACTAGAG
GCTGTATATGAAGCAATTTTTGGAAAGCCAAAGGAGAAGGTATACGCCGACGAGATA
GCAAAAGCCTGGGAAAGTGGAGAGAACCTTGAGAGAGTTGCCAAATACTCGATGGAA
GATGCAAAGGCAACTTATGAACTCGGGAAAGAATTCCTTCCAATGGAAATTCAGCTT
TCAAGATTAGTTGGACAACCTTTATGGGATGTTTCAAGGTCAAGCACAGGGAACCTT
GTAGAGTGGTTCTTACTTAGGAAAGCCTACGAAAGAAACGAAGTAGCTCCAAACAAG
CCAAGTGAAGAGGAGTATCAAAGAAGGCTCAGGGAGAGCTACACAGGTGGATTCGTT
AAAGAGCCAGAAAAGGGGTTGTGGGAAAACATAGTATACCTAGATTTTAGAGCCCTA
TATCCCTCGATTATAATTACCCACAATGTTTCTCCCGATACTCTAAATCTTGAGGGA
TGCAAGAACTATGATATCGCTCCTCAAGTAGGCCACAAGTTCTGCAAGGACATCCCT
GGTTTTATACCAAGTCTCTTGGGACATTTGTTAGAGGAAAGACAAAAGATTAAGACA
AAAATGAAGGAAACTCAAGATCCTATAGAAAAAATACTCCTTGACTATAGACAAAAA
GCGATAAAACTCTTAGCAAATTCTTTCTACGGATATTATGGCTATGCAAAAGCAAGA
TGGTACTGTAAGGAGTGTGCTGAGAGCGTTACTGCCTGGGGAAGAAAGTACATCGAG
TTAGTATGGAAGGAGCTCGAAGAAAAGTTTGGATTTAAAGTCCTCTACATTGACACT
GATGGTCTCTATGCAACTATCCCAGGAGGAGAAAGTGAGGAAATAAAGAAAAAGGCT
CTAGAATTTGTAAAATACATAAATTCAAAGCTCCCTGGACTGCTAGAGCTTGAATAT
GAAGGGTTTTATAAGAGGGGATTCTTCGTTACGAAGAAGAGGTATGCAGTAATAGAT
GAAGAAGGAAAAGTCATTACTCGTGGTTTAGAGATAGTTAGGAGAGATTGGAGTGAA
ATTGCAAAAGAAACTCAAGCTAGAGTTTTGGAGACAATACTAAAACACGGAGATGTT
GAAGAAGCTGTGAGAATAGTAAAAGAAGTAATACAAAAGCTTGCCAATTATGAAATT
CCACCAGAGAAGCTCGCAATATATGAGCAGATAACAAGACCATTACATGAGTATAAG
GCGATAGGTCCTCACGTAGCTGTTGCAAAGAAACTAGCTGCTAAAGGAGTTAAAATA
AAGCCAGGAATGGTAATTGGATACATAGTACTTAGAGGCGATGGTCCAATTAGCAAT
AGGGCAATTCTAGCTGAGGAATACGATCCCAAAAAGCACAAGTATGACGCAGAATAT
TACATTGAGAACCAGGTTCTTCCAGCGGTACTTAGGATATTGGAGGGATTTGGATAC
AGAAAGGAAGACCTCAGATACCAAAAGACAAGACAAGTCGGCCTAACTTCCTGGCTT
AACATTAAAAAATCCGGTACCGGCGGTGGCGGTGCAACCGTAAAGTTCAAGTACAAA
GGCGAAGAAAAAGAGGTAGACATCTCCAAGATCAAGAAAGTATGGCGTGTGGGCAAG
ATGATCTCCTTCACCTACGACGAGGGCGGTGGCAAGACCGGCCGTGGTGCGGTAAGC
GAAAAGGACGCGCCGAAGGAGCTGCTGCAGATGCTGGAGAAGCAGAAAAAGTAG

Pfu-Sso7d fusion-part with dU-defined overlaps indicated in bold:

--PfuR-----
…TCGGCCTAACTTCCTGGCTT

------------------dU-overlap------------Sso7d-1F/R-------
AACATTAAAAAATCCGGT**ACCGGCGGT**GGCGGTGCAACCGTAAAGTTCAAGTACAAA

------------------------------dU-overlap-----------------
GGCGAAGAAAAAGAGGTAGACATCTCCAAG**ATCAAGAAAGT**ATGGCGTGTGGGCAAG

------Sso7d-2F/R------------------dU-overlap-------------
ATGATCTCCTTCACCTACGACGAGGGCGGTGGCA**AGACCGGCCGT**GGTGCGGTAAGC

----------------------Sso7d-3F/R----------------------…
GAAAAGGACGCGCCGAAGGAGCTGCTGCAGATGCTGGAGAAGCAGAAAAAGTAG…

N-terminal His-tag sequence highlighted in yellow
Pfu sequence not high-lighted
Pfu-Sso7d linker sequence highlighted in green
Sso7d sequence highlighted in blue
