# Supplementary material for: A mutant Pfu DNA polymerase designed for advanced uracil-excision DNA engineering
Source: BMC Biotechnol. 2010 Mar 16;10:21. doi: 10.1186/1472-6750-10-21 (PMC2847956; doi:10.1186/1472-6750-10-21)
Supplement: Additional file 2 — Comparison of the DNA polymerases PfuTurbo, Phusion and PfuX7 in a site-directed mutagenesis PCR. Shows the comparison of PfuTurbo, Phusion (PfuS7) and PfuX7 in a site-mutagenesis PCR in pdf format. [file 1472-6750-10-21-S2.PDF]

Additional file 2

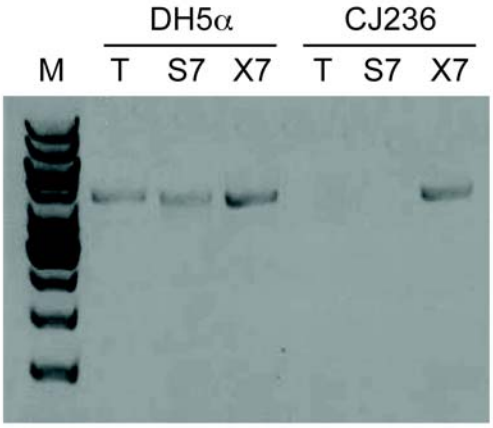

Additional file 2. Comparison of the DNA polymerases PfuTurbo (T), Phusion (S7), and PfuX7 (X7) in site-directed mutagenesis PCR. Agarose gel electrophoresis of PCRs performed with different DNA polymerases using template plasmid DNA isolated from the *ung+ E. coli* strain DH5α or the *ung-* strain CJ236. The molecular marker (M) is kb+ (Invitrogen).
